# New Perspectives on Polycythemia Vera: From Diagnosis to Therapy

**DOI:** 10.3390/ijms21165805

**Published:** 2020-08-13

**Authors:** Alessandra Iurlo, Daniele Cattaneo, Cristina Bucelli, Luca Baldini

**Affiliations:** 1 Hematology Division, Foundation IRCCS Ca’ Granda Ospedale Maggiore Policlinico, 20122 Milan, Italy; cattaneo.daniele@alice.it (D.C.); cbucelli@libero.it (C.B.); luca.baldini@unimi.it (L.B.); 2 Department of Oncology and Hemato-Oncology, University of Milan, 20122 Milan, Italy

**Keywords:** polycythemia vera, risk factors, target therapy, hydroxyurea, ruxolitinib, interferon, givinostat, idasanutlin

## Abstract

Polycythemia vera (PV) is mainly characterized by elevated blood cell counts, thrombotic as well as hemorrhagic predisposition, a variety of symptoms, and cumulative risks of fibrotic progression and/or leukemic evolution over time. Major changes to its diagnostic criteria were made in the 2016 revision of the World Health Organization (WHO) classification, with both hemoglobin and hematocrit diagnostic thresholds lowered to 16.5 g/dL and 49% for men, and 16 g/dL and 48% for women, respectively. The main reason leading to these changes was represented by the recognition of a new entity, namely the so-called “masked PV”, as individuals suffering from this condition have a worse outcome, possibly owing to missed or delayed diagnoses and lower intensity of treatment. Thrombotic risk stratification is of crucial importance to evaluate patients’ prognosis at diagnosis. Currently, patients are stratified into a low-risk group, in the case of younger age (<60 years) and no previous thromboses, and a high-risk group, in the case of patients older than 60 years and/or with a previous thrombotic complication. Furthermore, even though they have not yet been formally included in a scoring system, generic cardiovascular risk factors, particularly hypertension, smoking, and leukocytosis, contribute to the thrombotic overall risk. In the absence of agents proven to modify its natural history and prevent progression, PV management has primarily been focused on minimizing the thrombotic risk, representing the main cause of morbidity and mortality. When cytoreduction is necessary, conventional therapies include hydroxyurea as a first-line treatment and ruxolitinib and interferon in resistant/intolerant cases. Each therapy, however, is burdened by specific drawbacks, underlying the need for improved strategies. Currently, the therapeutic landscape for PV is still expanding, and includes several molecules that are under investigation, like long-acting pegylated interferon alpha-2b, histone deacetylase inhibitors, and murine double minute 2 (MDM2) inhibitors.

## 1. Introduction

Polycythemia vera (PV), together with essential thrombocythemia (ET) and myelofibrosis (MF), belongs to the so-called “classic” *BCR-ABL1*-negative myeloproliferative neoplasms (MPN), a heterogeneous group of diseases, characterized by the clonal expansion of an abnormal hematopoietic stem/progenitor cell. Its incidence has been estimated to be 2.3–2.8 per 100,000 persons/year, with a median age at diagnosis of about 60 years and a male/female ratio of 1.2:1 [1]. It is mainly characterized by elevated blood cell counts, especially red blood cells; thrombotic as well as hemorrhagic predisposition; a variety of symptoms; and cumulative risks of progression to MF and/or transformation over time into acute myeloid leukemia.

The understanding of MPN pathophysiology dramatically improved following the description of recurrent molecular abnormalities. In particular, compared with both ET and MF, PV is molecularly more homogeneous, being driven by *JAK2* mutations in virtually all cases [2]; about 97% of such mutations are represented by *JAK2*V617F, which results from a somatic G to T mutation involving *JAK2* exon 14, leading to a nucleotide change at position 1849 and the substitution of valine to phenylalanine at codon 617 [3]. *JAK2*V617F-negative PV occurs in 1–3% of patients and mostly involves *JAK2* exon 12 [4]. Mouse models and clinical studies have both revealed phenotypic differences between *JAK2* exon 12 and *JAK2*V617F-mutated PV, the former being characterized by erythroid-dominant myeloproliferation, subtler tri-lineage hyperplasia in the bone marrow (BM), and younger age [5,6]. Concerning the V617F allele burden, in MPN patients, it correlates with both hematologic characteristics and clinical features [7]. Specifically in PV, *JAK2*V617F homozygosity seems to be associated with a stimulated erythropoiesis and myelopoiesis, lower platelet count, a higher incidence of splenomegaly, a larger spleen size, and a greater proportion of patients requiring cytoreductive therapy, as well as with a higher incidence of pruritus. On the contrary, the rate of major thromboses is not increased in homozygous PV patients compared with heterozygous subjects [8,9]. More recently, other *JAK2* variants have been identified, with *JAK2*V625F and *JAK2*F556V being gain-of-function mutations [10].

Furthermore, *JAK2*V617F mutation has been proven to play a crucial role in thrombotic complications. In detail, the pathogenesis of blood clotting activation in this disease is multifactorial, and involves various anomalies of platelets, erythrocytes, and leukocytes, as well as dysfunction of endothelial cells [11]. Indeed, abnormalities of blood cells arising from the clonal hematopoietic stem cells’ proliferation also involve qualitative changes that characterize the switch of these cells from a resting to a procoagulant phenotype [12]. Prothrombotic features include blood cells’ expression of procoagulant and proteolytic properties, inflammatory cytokines secretion, and the expression of adhesion molecules. Specifically concerning platelets, different studies showed that, in MPN patients, they circulate in an activated status, as assessed by the detection of increased expression of surface P-selectin and tissue factor [13,14,15] and by the increased fraction of platelets phagocytosed by circulating neutrophils and monocytes [16]. In addition, the thrombin generation induced by platelets was found to be increased and associated with platelet activation, particularly in *JAK2*V617F-mutated cases [17]. Interestingly, immature platelets, the newly formed platelets that show a higher hemostatic activity [18], are more elevated and more reactive than their mature counterpart, and positively correlate with the presence of the *JAK2*V617F mutation [19]. With regards to red blood cells, an abnormal adhesion of the latter to the sub-endothelial protein laminin, owing to the phosphorylation of Lu/BCAM by *JAK2*V617F pathway, has been demonstrated [20]. Moreover, neutrophils play a crucial role in the inflammatory response and in the blood coagulation system activation [21]. In particular, the release of proteolytic enzymes (i.e., elastase, cathepsin G) and reactive oxygen species (ROS) and the increased expression of CD11b on their surface can activate/damage platelets and endothelial cells and impair some coagulation proteins [22,23]. The adhesion of platelets to leukocytes and the formation of platelet–leukocyte aggregates mediate the crosstalk between platelets, neutrophils, and monocytes [24], suggesting that aspirin may inhibit the interaction between neutrophils and platelets [13]. Finally, several factors, such as ROS and intracellular proteases, may perturb the physiological state of endothelium in MPN patients and turn it into a pro-adhesive and procoagulant surface [24].

Splenomegaly is estimated to affect 30% to 40% of PV patients and is usually associated with an advanced disease. In addition, spleen enlargement during follow-up has been shown to be significantly associated with an increased risk of fibrotic transformation and/or leukemic evolution [25,26].

Cytogenetic abnormalities can be detected in 14–20% of patients at the time of the initial diagnosis of PV [27,28,29,30], with del(20q), + 8, + 9, and + 1q being the most commonly reported [31,32,33]. The low frequency of abnormal karyotypes has made prognostication of PV patients using cytogenetic data challenging. While some studies have not shown a prognostic difference based on cytogenetic characteristics [27], other investigations, including one by the International Working Group for Myeloproliferative Neoplasms Research and Treatment (IWG-MRT), have recorded a higher risk of disease progression and a worse outcome in patients with an abnormal karyotype [28,34,35].

More recently, the occurrence and prognostic relevance of DNA sequence variants/mutations other than *JAK2/CALR/MPL* in PV were described [36,37,38]. In particular, a myeloid neoplasm-relevant 27-gene panel was used for next-generation sequencing (NGS) in 133 Mayo Clinic PV patients, revealing that 53% of them harbored one or more sequence variants/mutations other than *JAK2/CALR/MPL*; the most frequent were *TET2* and *ASXL1*. Adverse variants/mutations, in terms of overall (OS), leukemia-free (LFS), or myelofibrosis-free survival, included *ASXL1*, *SRSF2*, and *IDH2*, with a combined prevalence of 15%. The latter were also associated with an inferior OS (median, 7.7 versus 16.9 years) and their effect was independent of other conventional prognostic models. These observations were then validated in 215 Italian PV patients. In both the Mayo Clinic and Italian cohorts, leukemic or fibrotic progression, but not the thrombotic risk, was also predicted by adverse variants/mutations. Furthermore, the mutations number did not provide additional prognostic information [38].

On the basis of these data, the same group of authors have recently examined the possibility of integrating genetic information for predicting survival in PV. In a large cohort of 906 molecularly-annotated patients, including 404 with PV, adverse mutations occurred in 8 (2%) PV patients and multivariable analysis identified spliceosome mutations (*SRSF2*) to adversely affect OS. Furthermore, they also suggested the independent survival effect of adverse mutations, age >67 years, leukocyte count ≥15 × 10^9^/L, and previous thromboses in PV. A subsequent hazard ratio (HR)-based risk point allocation allowed the development of a three-tiered mutation-enhanced international prognostic system (MIPSS) whose performance was shown to be superior to other conventional scoring systems [39].

### 1.1. Diagnostic Criteria

Major changes to the PV diagnostic criteria were made in the 2016 revision of the World Health Organization (WHO) classification (Table 1) [2]. In particular, the diagnostic thresholds for hemoglobin (Hb) and hematocrit (Hct) were both lowered to 16.5 g/dL and 49% for men, and 16 g/dL and 48% for women, respectively. The dismissal of the endogenous erythroid colony formation “in vitro” as a minor diagnostic criterion should also be mentioned; indeed, although highly specific for *JAK2*V617F-mutated, erythropoietin-independent erythroid progenitors [40], it suffers from being technically demanding and expensive, and it is available only in a very limited number of research laboratories.

Furthermore, as we have previously reported, the use of the WHO 2016 revised diagnostic criteria led to a higher number of PV diagnosis among those cases that would have been formerly classified as MPN, unclassifiable owing to the lack of all required diagnostic criteria [41].

The main reason for these changes was represented by the recognition of a new entity, namely the so-called “masked PV”; indeed, individuals suffering from this condition have a worse outcome [42,43], possibly owing to missed or delayed diagnoses and, as a consequence, a lower intensity of treatment [44]. In addition, BM biopsy has been included among the major criteria for PV diagnosis; first of all, it can be helpful to distinguish between PV and *JAK2*-positive ET [43], and it also enables the assessment of BM fibrosis grade at diagnosis, thus identifying a more aggressive disease [45,46]. Indeed, increased BM reticulin fibrosis (>1) at diagnosis has been found in 20–51% of patients [47,48,49]. In a previous report by the IWG-MRT, mostly mild BM reticulin fibrosis (grade > 1 of a three-graded score system) at diagnosis was associated with both a lower risk of thrombosis during the clinical course and a higher risk of fibrotic progression, while OS or LFS were not affected [50]. In a more recent study, about 127 (48%) out of 262 PV patients displayed grade > 1 reticulin fibrosis at the time of diagnosis, without significant differences in presenting clinical and laboratory features. In univariate analysis, BM fibrosis had no significant impact on OS, LFS, or thrombosis-free survival, whereas a significant association was recorded for myelofibrosis-free survival (HR 2.9; 95% confidence interval (CI) 1.32–6.78, *p* = 0.009) [51].

### 1.2. Prognostic Stratification for Thrombosis

As thrombotic events represent the main cause of morbidity and mortality for PV patients, with a registered rate of cardiovascular (CV) deaths and non-fatal thrombotic events of 5.5% patients/year [52], CV risk stratification is of crucial importance to evaluate patients’ prognosis at diagnosis.

In the observational, prospective ECLAP study, which enrolled 1.638 PV patients, the global incidence of both arterial and venous thromboses was significantly higher among older patients (age > 65 years) or in the case of a previous thrombosis [52]. In a subsequent study involving 1.545 PV subjects, independent predictive factors for arterial thromboses included leuko-erythroblastosis, arterial hypertension, and previous arterial thrombotic events [34]. On the contrary, an abnormal karyotype and previous venous thromboses correlated with an increased risk of venous complications [53]. As a consequence, PV patients are currently stratified into two thrombotic risk classes: a low-risk group, in the case of younger patients (age < 60 years) with no previous thromboses, and a high-risk group, in the case of patients older than 60 years and/or with a previous thrombotic complications (Table 2).

Generic CV risk factors, particularly hypertension [54,59], smoking, and leukocytosis, while not yet formally included in a risk scoring system, contribute, as one can imagine, to the overall risk of thrombosis [60]. Hypertension was found to increase the annual rate of thrombosis from 0.85% patients/year to 2.05% patients/year and from 2.4% patients/year to 3.65% patients/year in the low- and high-risk conventional category, respectively. Regarding leukocytosis, in a time-dependent multivariable analysis of the ECLAP cohort [61], a leukocyte count >15 × 10^9^/L increased the HR of major thrombosis by 1.71-fold (95% CI, 1.1–2.6) compared with patients with ≤10 × 10^9^/L leukocytes [55]. Furthermore, in the prospective randomized CYTO-PV trial enrolling 365 patients with PV [62] and aimed at testing the effects of intensity of cytoreduction on thrombosis rate, a leukocyte count ≥11 × 10^9^/L accounted for an HR for thrombosis of 3.9 (95% CI, 1.24–12.03) compared with the reference value (leukocytes < 7 × 10^9^/L) [56].

Concerning other new possible CV risk factors, red cell distribution width (RDW) has been investigated and higher values might represent different pathophysiological processes, including excessive erythropoiesis, more active myeloproliferation, and subclinical inflammation favoring CV disease development in MPNs [58]. In addition, lymphocyte percentage (<17%) and RDW (<15%) might be predictors of vascular complications in patients without a history of thrombosis, and lymphocyte percentage (>13%) and platelet count (>393 × 10^9^/L) in the case of patients with a history of thrombosis [57].

### 1.3. Therapy

In the absence of agents proven to modify its natural history and prevent progression to advance phases, PV management has primarily been focused on minimizing the risk of thrombo-hemorrhagic complications that represent the major cause of morbidity and mortality [63,64]. Typical frontline management includes a combination of phlebotomy to decrease Hct to <45% and low-dose aspirin [61,62]. Underscoring the importance of a careful management, a study by Marchioli et al. found that treating to an Hct target range of 45–50% versus stringently maintaining it at <45% at a median follow-up of 31 months was associated with a fourfold increase in death due to CV adverse events (AEs) or major thromboses [62].

The anti-thrombotic efficacy and safety profile of low-dose aspirin in PV have been assessed in the ECLAP double-blind, placebo-controlled, randomized clinical trial [61]. In this study, 518 PV patients were randomized to receive aspirin 100 mg once daily or placebo. After a follow-up of about three years, low-dose aspirin reduced the combined risk of non-fatal cardio-embolic events or cardiovascular death by 60% (relative risk, 0.40; 95% CI 0.18–0.91, *p* = 0.0277), with no significant increase of major bleeding episodes (relative risk, 1.62; 95% CI 0.27–9.71).

Low-dose aspirin therapy was also effective in alleviating microvascular disturbances associated with PV [65], as these symptoms are believed to stem from small vessel-based abnormal platelet–endothelial interactions [66].

Furthermore, it was suggested that twice-daily aspirin may work better than once daily dose in certain cases [67]. Accordingly, such a therapeutic approach should be considered in patients who seem to be resistant to once daily dosing or considered at higher risk of arterial thrombosis [68].

Whether these results should be re-interpreted based on recent studies failing to demonstrate risk-balanced effectiveness of aspirin for primary prophylaxis in large cohorts of normal individual without prior history of atherosclerotic cardiovascular disease [69] represents an important research issue for future studies. In the meanwhile, considering that PV patients constitute a population of subjects at intrinsically high risk of CV events, according to the European LeukemiaNet (ELN) recommendations, low-dose aspirin (81 to 100 mg daily) should be used as primary anti-thrombotic prophylaxis for all PV patients with no major contraindications to aspirin, regardless of their risk categories [70].

Management of white blood cell (WBC) and platelet counts is also an important treatment goal because the risk of major thromboses was shown to be approximately four times greater in patients with WBC counts ≥ 11 × 10^9^/L versus <7 × 10^9^/L (*p* = 0.02) [56]. On the basis of these data, in order to achieve Hct target levels and to normalize WBC and platelet counts according to the ELN recommendations, many patients require a cytoreductive treatment [70,71,72]. For this purpose, conventional therapies include hydroxyurea (HU) as first-line option, ruxolitinib and interferon (IFN) in resistant/intolerant cases, and busulfan in older subjects. Each of these drugs, however, is burdened by specific drawbacks underlying the need for improved therapeutic strategies.

Currently, the therapeutic landscape for PV is expanding. Novel agents are in development with the aim not only to reduce the thrombotic potential, but also to act directly on the malignant clone with the aim of significantly modifying disease progression. Among these, long-acting pegylated interferon (PEG-IFN) alpha-2b, histone deacetylase inhibitors, and MDM2 inhibitors should be mentioned (Table 3).

### 1.4. Hydroxyurea

Hydroxyurea is among the most commonly used cytoreductive treatments for PV; however, some patients may not achieve an adequate benefit with persistent disease-related manifestations or may not tolerate long-term therapy [73,74]. Indeed, a study by Alvarez-Larrán et al. found that 12 and 13% of patients had resistance or intolerance to HU, respectively, with the former significantly associated with an increased risk of death and the latter altering quality of life [75]. These data further denote a significant medical need for some patients with PV currently or previously treated with HU.

Furthermore, in a more recent study from the same group, the probability of developing resistance after 5 years of HU treatment was 64% in patients with *TP53* disruption/aneuploidy, 49% with spliceosome or chromatin aberrations, 27% with homozygous *JAK2* mutation, and 14.5% with heterozygous *JAK2* mutation (*p* < 0.0001 for comparison between groups). In multivariate analysis, genomic classification was associated with risk of resistance to HU (HR 2.2; 95% CI: 1.5–3.2, *p* < 0.0001) after correction for age (HR 1.01; 95% CI: 0.97–1.03, *p* = 0.9), sex (HR 0.7; 95% CI: 0.9–1.2, *p* = 0.4), Hb value (HR 1.08; 95% CI: 0.9–1.2, *p* = 0.3), WBC count (HR 1.1; 95% CI: 1.01–1.18, *p* = 0.02), platelet count (HR 1.0; 95% CI: 0.9–1.002, *p* = 0.9), and spleen size (HR 1.03; 95% CI: 0.9–1.2, *p* = 0.7) [76].

### 1.5. Ruxolitinib

The use of the *JAK1/2* inhibitor ruxolitinib in PV was approved by the FDA in 2014 for patients with an inadequate response to or an unacceptable toxicity from HU. It was initially evaluated in a phase II trial in 34 patients with advanced PV refractory or intolerant to HU. At the dosage of 10 mg twice daily, a rapid and durable clinical benefit in terms of reduction of Hct, resolution of splenomegaly, normalization of WBC, and platelet counts, and improvement of PV-associated symptoms was observed [77]. The phase III open-label RESPONSE trial was thus designed in order to evaluate the efficacy and safety of ruxolitinib versus standard therapy (BAT) in 222 phlebotomy-dependent patients who were assigned to receive either ruxolitinib or BAT. The primary endpoint, defined as the proportion of patients who achieved both Hct control and a reduction of at least 35% in spleen volume at week 32, was obtained in 21% of patients in the ruxolitinib group versus 1% in the BAT group. Hematocrit control (60% versus 20%) and spleen volume reduction (38% versus 1%) both favored the ruxolitinib group. A better control of PV symptoms was also recorded in the ruxolitinib arm. Besides grade 3/4 anemia and thrombocytopenia (2% and 5%, respectively), Herpes Zoster reactivation was the most common infectious complication, being reported in 6% of ruxolitinib-treated subjects [78]. At 80 weeks, 83% of patients randomized to ruxolitinib remained on therapy, while 88% of the BAT group underwent crossover. The probability of maintaining the primary endpoint and a complete hematological response (CHR) for ≥80 weeks was 92% versus 69% in the ruxolitinib and BAT group, respectively. A thromboembolic event rate of 1.8 versus 8.2 per 100 patients/year, respectively, was also reported. In the ruxolitinib arm, a higher risk for non-melanoma skin cancer was observed [79]. At 5 years of follow-up, 98% of patients initially assigned to BAT crossed over to ruxolitinib, with a probability of survival at 5 years of 91.9% with ruxolitinib and 91.0% with BAT [80]. In the RESPONSE-2 open-label phase IIIb study, efficacy and safety of ruxolitinib versus BAT were assessed in 149 PV patients without palpable splenomegaly resistant or intolerant to HU; BAT included HU (49% of subjects) and IFN or PEG-IFN (13% of subjects). The primary endpoint, namely Hct control at week 28, was achieved by 62% versus 19% of patients in the ruxolitinib and BAT group, respectively. No cases of grade 3/4 anemia or thrombocytopenia occurred with ruxolitinib, while in the BAT group, they were reported in one and three subjects, respectively [81]. At week 80, 93% of subjects randomized to ruxolitinib were receiving this treatment, while no patient remained on BAT. At the same time-point, a durable CHR was achieved in 24% of patients in the ruxolitinib arm versus 3% in the BAT arm [82].

In the double-blind phase IIIb RELIEF trial, 110 PV patients with an adequate Hct control on HU, but with PV-related symptoms were randomized either to receive ruxolitinib or continue HU. The primary endpoint, that is, the proportion of patients achieving a ≥50% reduction from baseline in total symptom score (TSS) at week 16, was not significantly superior in the ruxolitinib group (43.4% versus 29.6%). However, in a post-hoc analysis, a statistically significant improvement in the primary endpoint was observed in patients with stable TSS from screening to baseline [83].

Interestingly, in a recent meta-analysis that considered four randomized controlled trials, including 663 patients (1057 patients/year), the number of thrombotic events reported with ruxolitinib was consistently lower than that with BAT, but, globally, the difference did not reach significance (*p* = 0.098) [84].

On the basis of findings suggesting that ruxolitinib increases the efficacy and tolerability of PEG-IFN alpha-2, a phase II trial investigated the combination of ruxolitinib with low-dose PEG-IFN alpha-2 in 32 patients with PV and 18 with MF previously intolerant or refractory to PEG-IFN alpha-2. As far as PV subjects are concerned, 9% achieved a complete and 22% a partial remission; in addition, the median *JAK2*V617F burden decreased from 47% to 12%. Out of 36 patients previously intolerant to PEG-IFN alpha-2, 86% completed the study with acceptable toxicity [85].

### 1.6. Interferon

For several decades, IFN-alpha has been used to control erythrocytosis in most patients with PV. A similar efficacy has also been observed in terms of reduction of spleen size or relief from pruritus. Interferon-alpha has proved to be a useful treatment option, because, differently from conventional therapeutic approaches, it is devoid of pro-leukemic effect [86,87,88].

Nowadays, novel formulations of this drug, such as PEG forms, are making their way into the clinical practice because of an even better safety profile [89]. Furthermore, even though the literature on PV is scarcer than on ET, IFN-alpha still represents the only safe option to be used during pregnancy owing to the absence of any teratogenic effect [90,91].

### 1.7. Ropeginterferon Alpha-2b

Ropeginterferon alpha-2b (BESREMI, Pharma Essentia) was approved by EMA on February 2019 as monotherapy for the treatment of PV patients without symptomatic splenomegaly. It is a mono-PEG-IFN alpha-2b characterized by higher tolerability and longer half-life than conventional PEG-IFN alpha. In a multicenter phase I/II clinical trial aimed at defining Ropeginterferon alpha-2b safety and efficacy, complete and partial hematological response were 47% and 43%, respectively, and drug-related AEs were those usually associated with IFN-alpha administration [92]. On the basis of these encouraging findings, the PROUD-PV and its extension CONTINUATION-PV clinical trials were performed in order to compare Ropeginterferon alpha-2b with HU. The drug was administered subcutaneously at a starting dose of 45 µg weekly and titrated monthly in 45 µg increments for response up to a maximum of 180 µg weekly. Dose escalation occurred when the criteria for CHR and dose-limiting toxicity were not met. At 12 months in the PROUD-PV study, the primary endpoint (i.e., non-inferiority of Ropeginterferon alpha-2b versus HU in terms of CHR with normalization of spleen size) was achieved by 21% of patients in the Ropeginterferon alpha-2b group and by 28% of those in the HU group, while CHR without spleen normalization was recorded in 43% versus 46% of subjects, respectively. At an interim analysis at 36 months in the CONTINUATION-PV study, CHR with improved disease burden was recorded in 53% of patients in the Ropeginterferon alpha-2b group versus 38% of those in the HU group; CHR without the spleen criterion was obtained in 71% versus 51% of the patients, respectively. At 24 and 36 months in the Ropeginterferon alpha-2b group, the mean *JAK2*V617F allele burden was significantly lower. Hepatic enzyme elevations in the Ropeginterferon alpha-2b group and cytopenias in the HU group were the most frequent grade 3/4 AEs; treatment-related psychiatric disorders were reported in 4 of 127 subjects treated with Ropeginterferon alpha-2b [93].

More recently, this IFN formulation has also been evaluated in the setting of low-risk PV patients enrolled in the low-PV phase II, investigator driven randomized trial (NCT030030025). In this study, a stringent and rigorous monthly phlebotomy policy (standard arm) was compared with Ropeginterferon alpha-2b administered subcutaneously every 2 weeks in a dose of 100 µg, on top of standard therapy. The primary endpoint, that is, the maintenance of Hct target, was reached in 84% of cases on Ropeginterferon versus 60% in the standard arm (*p* = 0.008). Concerning safety, no statistical difference was highlighted in grade 3 or more AEs, which accounted for 6% and 8% of patients treated with Ropeginterferon or standard therapy, respectively [94].

### 1.8. Givinostat

Histone deacetylases (HDACs) catalyze the removal of acetyl groups from lysine residues of histones, leading to down-regulation of tumor suppressor gene expression; because hypoacetylation has been detected in various hematological and solid malignancies, HDAC inhibitors are intensively investigated as anticancer agents [95]. Givinostat (Italfarmaco), an HDAC inhibitor, was initially evaluated “in vitro” on *JAK2*V617F-positive cells from PV and ET patients as well as on the erythroleukemia cell line HEL. By suppressing the clonogenic activity of mutated cells, givinostat allowed an outgrowth of unmutated colonies, thus demonstrating a specific inhibition of *JAK2*V617F protein and of its downstream signaling [96]. The efficacy and safety of givinostat at 50 mg daily were evaluated in a phase II study in 29 *JAK2*V617F-positive MPN patients, 12 of whom had PV. In this cohort, one complete and six partial hematological responses were achieved, with seven patients becoming independent of phlebotomy. Reduction of splenomegaly and pruritus as well as of the *JAK2*V617F allele burden was also reported. Furthermore, therapy was well tolerated, and no grade 4 toxicities were recorded [97]. In a randomized phase II study in subjects with *JAK2*V617F-positive PV unresponsive to HU monotherapy at maximum tolerated dose (MTD), 44 patients were randomized to givinostat 50 mg once daily or twice daily in combination with HU at MTD. In the 50 mg and 100 mg groups, complete plus partial responses were recorded in 55% versus 50% of subjects and control of pruritus in 64% versus 67% of patients, respectively. Grade 3 AEs were reported only in 4.5% of cases in each arm [98]. In order to allow PV patients who achieved a clinical benefit with givinostat to continue this treatment, a multicenter clinical trial aimed at evaluating its long-term efficacy and safety was performed. Forty-five subjects were recruited and treated for a median of 4 years. Complete and partial hematological remissions were observed in 11% and 89% of patients, respectively. Fifty-six percent of subjects had normal Hct without phlebotomy as well as a normal spleen size either by palpation or imaging; the overall incidence of thrombosis was 2.30% patients/year. Givinostat long-lasting therapy was well tolerated; only three grade 3 toxicities were reported, and no grade 4 AEs occurred [99]. A recent phase Ib/II study was designed in order to determine the MTD of givinostat as monotherapy and to assess safety and efficacy of this dose. Twelve PV subjects were recruited in the phase Ib part that identified 100 mg twice daily in 4-week cycles as the recommended dose for the phase II part. Thirty-five patients were enrolled; at the end of cycles 3 and 6, the overall response rate was 80.6%. Modest improvements were also recorded in *JAK2*V617F allele burden. Givinostat was well tolerated without safety concerns, as only two drug-related grade 3 AEs were recorded [100].

### 1.9. Idasanutlin

The murine double minute 2 (MDM2) is a negative regulator of p53, a tumor suppressor protein frequently inactivated in human cancers that controls crucial pathways protecting cells from malignant transformation [101,102]. In “ex vivo” cultures, MDM2 was found to be overexpressed in primary *JAK2*V617F-positive cells and a reduced p53 response to DNA damage was observed in CD34 +-derived erythroid progenitors from patients with *JAK2*V617F-positive MPNs. Cell treatment with nutlin-3, a selective small-molecule antagonist of MDM2, reactivated the p53 response [103]. When combined with low-dose PEG-IFN alpha-2a in “in vitro” cultures, nutlin-3 led to increased p53 levels and apoptosis in PV CD34 + cells [104]. Treatment of PV and MF CD34 + cells with RG7112, a more active nutlin, and PEG-IFN alpha-2a before transplantation into immune-deficient mice decreased the degree of donor-derived chimerism as well as the *JAK2*V617F allele burden, suggesting a depletion of MPN hematopoietic stem cells [105]. On the basis of these promising findings, a phase I study was activated on idasanutlin (Hoffmann-La Roche), a second-generation oral nutlin, given alone or in combination with IFN-alpha in patients with high-risk PV/ET for whom at least one prior line of therapy had failed. Twelve patients with *JAK2*V617F-positive PV/ET (11 and 1, respectively) were treated at two dose levels, 100 mg and 150 mg daily, for 5 consecutive days in a 28-day cycle, and those not achieving at least a partial response after six cycles were allowed to receive low-dose PEG-IFN alpha-2a. The response rate after six cycles of idasanutlin was 58% as a single agent and 50% in combination therapy, with an overall response rate of 75% and a median duration of response of 16.8 months. A decrease in the median number of phlebotomies in the 12 months following enrollment as well as reduction in TSS and splenomegaly resolution were recorded. A 43% median reduction in *JAK2*V617F variant allele frequency was also reported. Idasanutlin was well tolerated without dose-limiting toxicities. Five subjects (41.7%) experienced grade 3 non-hematologic AEs and grade 1/2 nausea occurred in 10 (83%) patients [106]. Idasanutlin is currently evaluated in a phase II trial (NCT03287245).

## 2. Conclusions

Arterial and/or venous thromboses represent the main cause of morbidity and mortality in PV. Therefore, treatment should be mainly focused on reduction of thrombotic risk, myeloproliferation control, improvement of symptomatic burden, and management of disease-associated complications. As has already happened for other hematological malignancies, as well as for PV, the goal is nowadays represented by patient-tailored therapies. While new drugs have recently entered the clinical arena with the promise to improve overall patients’ management, the evidence of a disease-modifying treatment is largely missing. There are several aspects concerning PV, however, that once clarified, might represent significant steps forward for a more adequate and successful treatment strategy, such as a better use of mutational genetics to improve the thrombotic risk stratification of PV patients and a more precise definition of response/safety criteria, which should guide the physician in selecting the most appropriate drug.

## Figures and Tables

**Table 1:** Diagnostic criteria for polycythemia vera according to the World Health Organization (WHO) classification.

	2008 WHO Classification	2016 WHO Classification
Major Criteria	1. Hb > 18.5 g/dL in men/Hb > 16.5 g/dL in women or other evidence of increased RCM; 2. Presence of *JAK2*V617F or other functionally similar mutation such as *JAK2* exon 12 mutation	1. Hb > 16.5 g/dL in men/Hb > 16.0 g/dL in women, or Hct > 49% in men/Hct > 48% in women, or increased RCM; 2. BM biopsy showing hypercellularity for age with trilineage growth (panmyelosis) including prominent erythroid, granulocytic, and megakaryocytic proliferation with pleomorphic, mature, megakaryocytes (differences in size); 3. Presence of *JAK2*V617F or *JAK2* exon 12 mutation
Minor Criteria	1. BM biopsy showing hypercellularity for age with trilineage growth (panmyelosis) with prominent erythroid, granulocytic, and megakaryocytic proliferation; 2. Subnormal serum EPO level; 3. Endogenous erythroid colony formation in vitro	Subnormal serum EPO level
Criteria required for diagnosis	All 2 major and 1 minor or the first major and 2 minor criteria	All 3 major or the first 2 major and the minor criterion

Abbreviations: Hb: hemoglobin; Hct, hematocrit; RCM, red cell mass; BM, bone marrow; EPO, erythropoietin.

**Table 2:** Risk factors for thrombosis in polycythemia vera patients: currently used and proposed ones.

	Risk Factors	
Currently Used	Low-risk	High-risk
	Age < 60 years [52] and no previous thrombosis [52]	Age > 60 years [52] and/or previous thrombosis [52]
Proposed	Hypertension [54] Smoking habit Leukocytosis (>15 × 10^9^/L [55] or >11 × 10^9^/L [56])	
Emerging	Platelet count [57] Abnormal karyotype [53] RDW [57,58] Lymphocyte percentage [57] Leuko-erythroblastosis [34]	

Abbreviations: RDW: red cell distribution width.

**Table 3:** Therapeutic landscape for polycythemia vera.

	Drug	Dosage
Approved	Hydroxyurea	0.5–2 g/day
	Ruxolitinib	10 mg twice daily
	Interferon-alpha	500,000–1 million units, 3 times weekly, progressively increased to 2–3 million units, 3 times weekly
	Ropeginterferon alpha-2b	starting dose of 45 µg weekly and titrated monthly in 45 µg increments up to a maximum of 180 µg weekly
Under development	Givinostat	100 mg twice daily
	Idasanutlin	100 or 150 mg daily, for 5 consecutive days in 28-day cycles
